# GEN: highly efficient SMILES explorer using autodidactic generative examination networks

**DOI:** 10.1186/s13321-020-00425-8

**Published:** 2020-04-10

**Authors:** Ruud van Deursen, Peter Ertl, Igor V. Tetko, Guillaume Godin

**Affiliations:** 1 Firmenich SA, Research and Development, Rue des Jeunes 1, Les Acacias, 1227 Geneva, Switzerland; 2 Novartis Institutes for BioMedical Research, Novartis Campus, 4056 Basel, Switzerland; 3 Institute of Structural Biology, Helmholtz Zentrum München-German Research Center for Environmental Health (GmbH), Ingolstädter Landstraße 1, 85764 Neuherberg, Germany; 4 BIGCHEM GmbH, Valerystr. 49, 85716 Unterschleißheim, Germany

**Keywords:** Autonomous learning, GEN, GAN, RNN, LSTM, GRU, biLSTM, biGRU, AI, SMILES, Generator, Quality control, SQC

## Abstract

Recurrent neural networks have been widely used to generate millions of de novo molecules in defined chemical spaces. Reported deep generative models are exclusively based on LSTM and/or GRU units and frequently trained using canonical SMILES. In this study, we introduce Generative Examination Networks (GEN) as a new approach to train deep generative networks for SMILES generation. In our GENs, we have used an architecture based on multiple concatenated bidirectional RNN units to enhance the validity of generated SMILES. GENs autonomously learn the target space in a few epochs and are stopped early using an independent online examination mechanism, measuring the quality of the generated set. Herein we have used online statistical quality control (SQC) on the percentage of valid molecular SMILES as examination measure to select the earliest available stable model weights. Very high levels of valid SMILES (95–98%) can be generated using multiple parallel encoding layers in combination with SMILES augmentation using unrestricted SMILES randomization. Our trained models combine an excellent novelty rate (85–90%) while generating SMILES with strong conservation of the property space (95–99%). In GENs, both the generative network and the examination mechanism are open to other architectures and quality criteria.
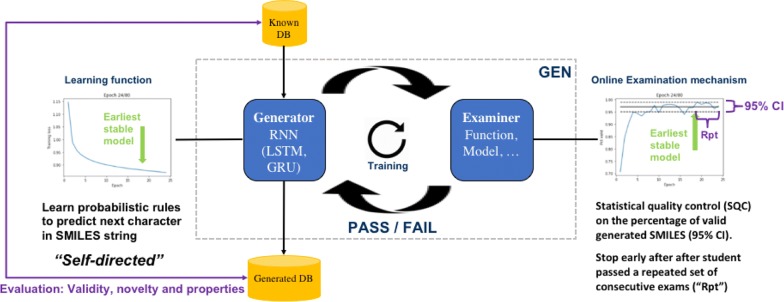

## Introduction

Exploration of chemical space for the discovery of new molecules is a key challenge for the chemical community, e.g. pharmaceutical and olfactive industries [1, 2]. Previously, exhaustive enumeration has been introduced with the creation of 26 M, 1G and 1.7G molecules in the databases GDB11, GDB13 and GDB17, respectively [3]. Exhaustive enumeration critically depends on knowledge rules specified by chemists to restrict the combinatorial explosion of possible molecules. Consequently, exhaustive enumeration may generate a realistic but more biased chemical space. More recently, AI methods have been emerging rapidly and have proven successful for text learning [4] and applications in drug discovery [5]. Deep generative models based on the SMILES syntax were reported as highly successful for the discovery of new molecules [6]. A recent publication shows that the architecture with a classical recurrent network introduces a bias to the generated space. These results were confirmed in a recently published work on GDB13, showing that about 68% of GDB-13 [3] was reproduced using a deep generative model [7]. SMILES [8] is a very simple text representation of molecules. It is “readable” by chemists and is quickly translated into molecules with RDKit [9] or other cheminformatics toolkits. Other 1D string encoders like InChI [10] or DeepSmiles [11] were reported with lower performance in deep generative models [3, 12]. Since 2016, SMILES-based machine-learned methods are used to produce de novo molecules. These methods include Variational AutoEncoders (VAEs) [12], Recurrent Neural Network (RNN) [6, 13–15], Generative Adversarial Networks (GANs) [16] and reinforcement learning (RL) [17] or generate molecules based on molecular graph representation [18] as well as other many approaches as reviewed by [19]. Contrary to these earlier reports, we demonstrate herein that text learning on SMILES is highly efficient to explore the training space with a high degree of novelty. Herein we have modified a previously reported algorithm [6] and use bidirectional RNN layers for better generation results. The neural network of the generator is subsequently converted into a generative examination network (GEN). In GENs, the deep generative models autonomously learn to write valid molecular SMILES. GENs are thus free to extract text-based rules to reconstruct the chemical space without being subjected to expert constraints. During training of the models, the learning progress of the generators is periodically examined using an independent online examination mechanism without feedback to the learning rate of the student. In this GEN we use an online generator that applies a statistical quality control after every training epoch, measuring the percentage of validity for a statistical set of generated SMILES. This mechanism is an early stopping function and prevents the network from overfitting [20] the training set to keep the highest degree of generativity. In GENs, the generator and examination methods are open to any other generative network and examination methods, including simple metrics or more advanced models. Our calculations based on the publicly available dataset PubChem [21], clearly demonstrate that the use of bidirectional layers systematically improves the capability of the GEN to generate a vast set of new SMILES within the property space of the training set. Following excellent results of SMILES augmentation for smaller datasets to predict physico-chemical properties [22–24] and generators [25], we have used SMILES augmentation to increase both the number and diversity of SMILES in the training set.

## Methods

### Preparation of datasets and encoding

The PubChem database was downloaded in March 2019 as SDF. The canonical SMILES string *PUBCHEM_OPENEYE_CAN_SMILES* was extracted, split into fragments and converted into canonical SMILES using RDKit version 2019.03.3 [9, 26]. Only organic molecules, i.e. those that contain at least one carbon and all other atoms are a subset of {H, B, C, N, O, F, S, Cl, Br or I} were retained. The remaining organic SMILES were de-duplicated to produce a set of unique SMILES. From this dataset, we extracted a representative set of 225k fragment-sized molecules typically explored in the pharmaceutical and olfactive industries [6, 27]. Prior to training, the SMILES were either converted to the canonical form or augmented as detailed in the results. Double character atoms were replaced by single characters: The characters Cl, Br and [nH] were modified to L, R and A, respectively. Stereochemistry was removed, replacing [C@H], [C@@H], [C@@] and [C@] by C as well as removing the characters/and\ used for double bond stereochemistry. The molecules were tokenized by making an inventory of observed characters followed by decoding the molecules. The generated text corpus was converted to a training set pairing the next available characters (labels) to the previously observed sentence, which were presented as one-hot encoded feature matrices to the network.

### Architecture

**Fig. 1:**
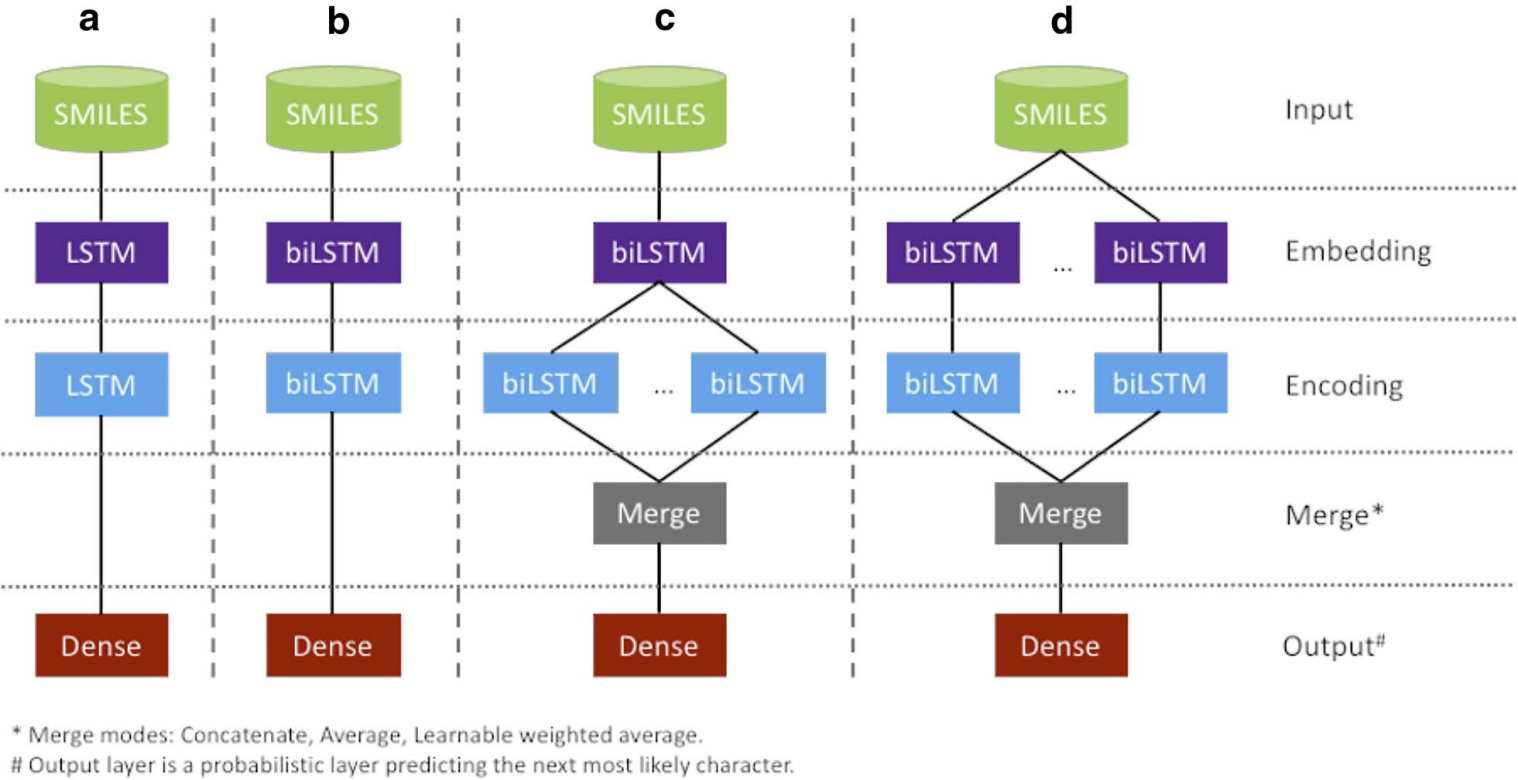
Tested architecture for SMILES generation. Architecture with two consecutive biLSTM layers used for deep-generative models for SMILES generation. **a** Original architecture with two consecutive LSTM layers, followed by a Dense output layer to predict the next character. **b** Modified architecture with two consecutive bidirectional LSTM layers. **c** Advanced architecture with one embedding biLSTM layers followed by multiple parallel bidirectional encoding layers and a merging layer (concatenated, averaged or learnable average). **d** Advanced architecture using parallel-concatenated architectures with multiplication of embedding and encoding layers. These layers are merge by concatenation, averaging or learnable weighted average

Modeling was performed using the open source libraries Tensorflow [28] and Keras [29]. The method was programmed in Python [30] and code is freely available [31] under a clause-3 BSD license. Architectures used for GENs were composed of an embedding biLSTM- or LSTM-layer, followed by a second encoding biLSTM- or LSTM-layer, a dropout layer (0.3) and a dense layer to predict the next character in the sequence (Fig. 1). For Architecture A and B, we also tested biGRU and GRU-layers for embedding and encoding. For consistency of the architecture, LSTM and GRU units were not mixed. Several runs were evaluated to reduce the set of hyperparameters. Here we have evaluated LSTM and GRU units with layer sizes of 64 and 256. The Dense layer had a size equal to the number of unique characters observed in the training set. Architectures C and D with multiple parallel encoding layers were evaluated using merging by concatenation, averaging or learnable weighted average (Fig. 1). The code for the layer of the learnable weighted average can be downloaded [31].

### Training of architecture with on-line statistical quality control

**Fig. 2:**
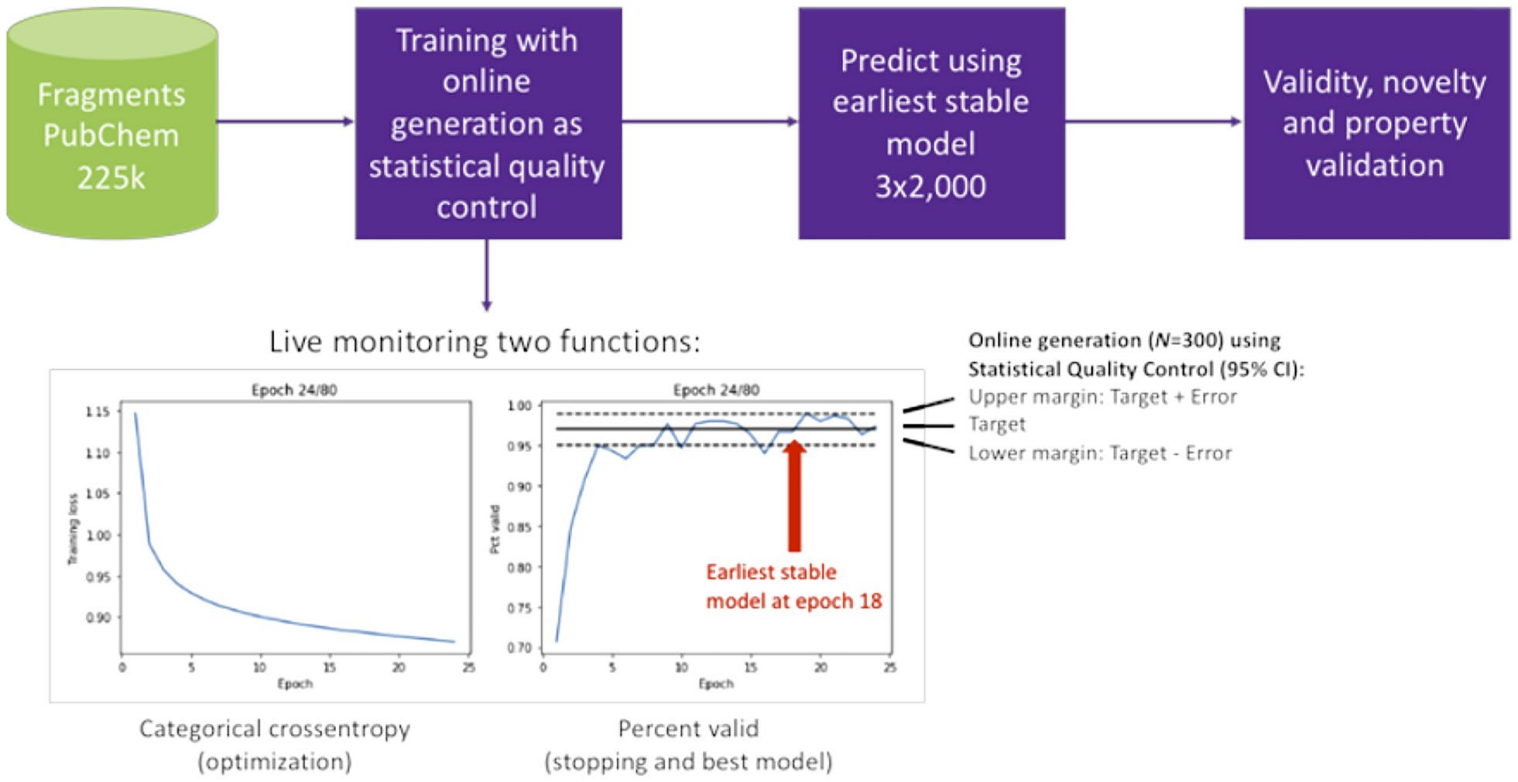
Modeling workflow used for every architecture/hyperparameter search. The autodidactic generator models learn independently a probability for the next logical character. At every epoch, i.e. online the generator generates a statistical sample of 300 SMILES Strings with are examined using statistical quality control as examination criteria. Upon completion of the training, the earliest stable model that satisfies the quality criteria is selected and evaluated based on a generated sample

It is widely known that LSTM is based on conservative long-range memory. Architectures A and B produced mostly canonical SMILES (> 92%) when trained with a set of canonical SMILES [32]. In order to improve the explorative nature of the GENs, we used a set of randomized SMILES with varying levels of augmentation. Early stopping was used to avoid overfitting and memorization of the training set [20]. In neural networks based on Keras, early stopping is applied using Callback functions (keras.callbacks.Callbacks). In our workflow (Fig. 2), we have modified the existing EarlyStopping function to generate a small sample of generated SMILES to measure the number of valid SMILES at every epoch [33]. On training start, the Callback function was parameterized with a target percentage, e.g. 97%, along with the sample size (***Nsample ***= 300). Optionally, the size of the population (**Npop**) was specified in the callback method. If no value *Npop* was specified, the size of the population is assumed to be very large i.e. ***N****pop* ≫ ***N****sample*. Based on the specified parameters, the upper and lower margins were computed using a 95% confidence interval (CI) [34]. The callback function stopped training early, if the trained model showed stable generation results within the 95% CI for 10 consecutive epochs to exclude incidental bad results (variable *patience*) [35]. The EarlyStopping counter was reset if the percentage of valid structures fell below the lower margin of the computed stability interval. Upon completion of training, the earliest available model was selected and used to generate 2k SMILES strings for evaluation. All evaluations were performed using three independently trained models and reported as average ± standard deviation. To have an objective assessment of quality, SMILES with easy-detectable errors, i.e. a mismatch for ring and branch closure characters, were included in the evaluation.

### Percentage of valid molecules, uniqueness and training compounds

For all generated molecules, the molecules were considered as valid if they were successfully converted to an InChIKey using RDKit [9, 10]. The percentage of valid molecules, Validity%, was measured as the ratio of the number of valid molecules to the number of generated SMILES. All valid molecules were reduced to unique compounds. The uniqueness, Uniqueness%, was expressed as a number of unique molecules divided by the number of valid molecules. The percentage of known training compound, Training%, was computing dividing the number of generated SMILES known in the training set, by the number of unique SMILES.

### Property distributions and percentage match

**Fig. 3:**
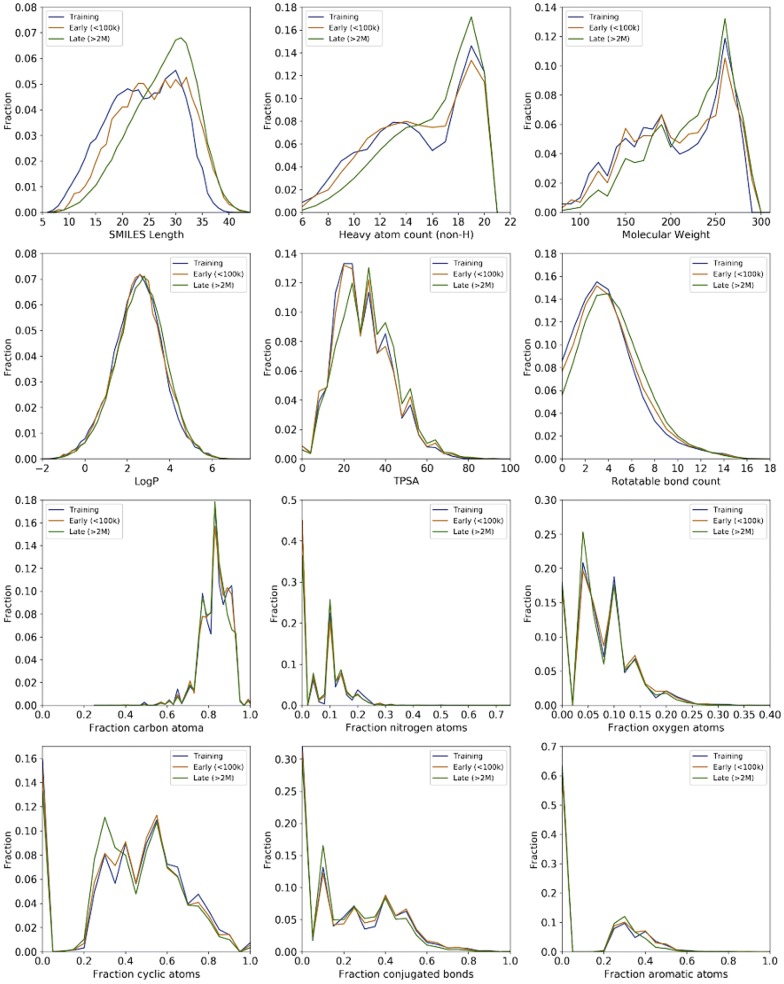
Property distributions for analyzed properties including size, topology, polarity and atom compositions. The data are shown for the training set (blue), early generation (orange) and late generation (green). The observed property shifts are due to the saturation for generation of molecules with smaller sizes (see also Fig. 4)

For the model with architecture C (biLSTM–biLSTM 256/256 with four concatenated encoding layers), 200 sets of 10k molecules were generated to create 2M molecules. For three sets of 10 k molecules at the beginning (early) and end (late) of generation process, we calculated property distributions for a set of 12 properties and compared to the property distributions of the training set (Fig. 3). Four classes of properties have been evaluated: A size comparison was performed using SMILES length (measured as number of characters), heavy atom count (HAC, counting all non-hydrogen atoms) and molecular weight; Polarity was evaluated using LogP and TPSA; Topological properties were compared using the number of rotatable bonds, fraction of cyclic, conjugated or aromatic atoms; A comparison on elemental composition was performed based on fractions of carbon, nitrogen or oxygen atoms in the molecules. All distributions are displayed in Fig. 3 and the percentage match for the distributions of the generated space *A* and training space ***B*** was computed using the continuous Tanimoto coefficient ***T(A,B)*** Eq. 1 [36].

1 $$T\left( {A,B} \right) = \frac{{\sum\nolimits_{i} {A_{i} B_{i} } }}{{\sum\nolimits_{i} {A_{i}^{2} } + \sum\nolimits_{i} {B_{i}^{2} } - \sum\nolimits_{i} {A_{i} B_{i} } }} \times 100\%$$

The Jensen-Shannon divergence ***JSD(A,B)*** between the normalized distributions ***A*** and ***B*** was computed applying Eq. 2: [37, 38].

2 $$\varvec{JSD}\left( {\varvec{A},\varvec{B}} \right) = \varvec{H}\left( {\sum\limits_{{\varvec{d} \in \left\{ {\varvec{A},\varvec{B}} \right\}}} {\varvec{a}_{\varvec{i}} \varvec{d}_{\varvec{i}} } } \right) - \left( {\sum\limits_{{\varvec{d} \in \left\{ {\varvec{A},\varvec{B}} \right\}}} {\varvec{a}_{\varvec{i}} \varvec{Hd}_{\varvec{i}} } } \right)$$

### Novelty analysis

**Fig. 4:**
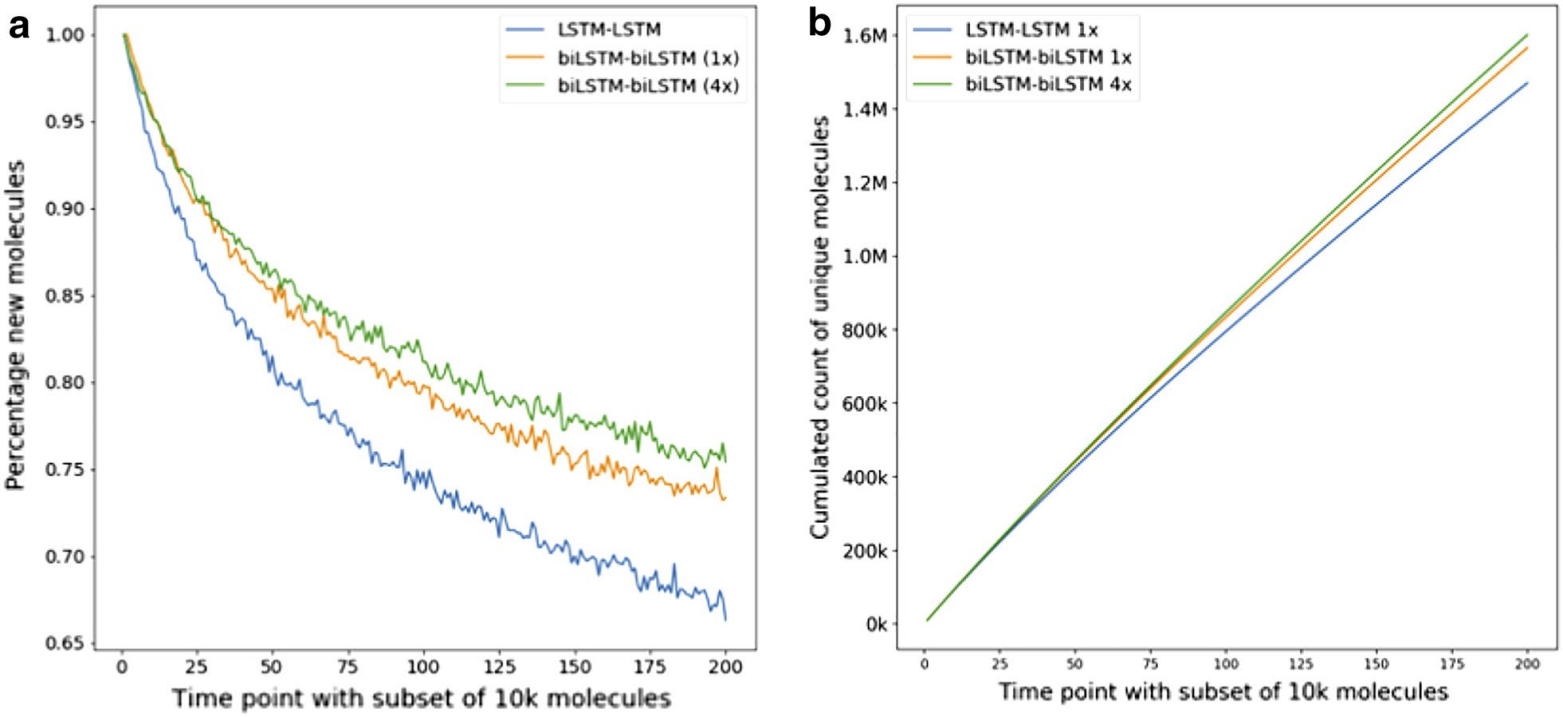
Global novelty analysis. For all sets of 2M generated compounds, the dataset has been split into 10k time points. **a** Plot showing the percentage of molecules at every time point t. **b** Cumulated number of unique molecules generated during the process. The final values for the three tested architectures are 1470,543 (73.5% efficiency) for LSTM–LSTM, 1566,535 (78.3% efficiency) for biLSTM–biLSTM and 1602,018 (80.1% efficiency) for biLSTM–biLSTM with 4 parallel-concatenated encoding layers

**Fig. 5:**
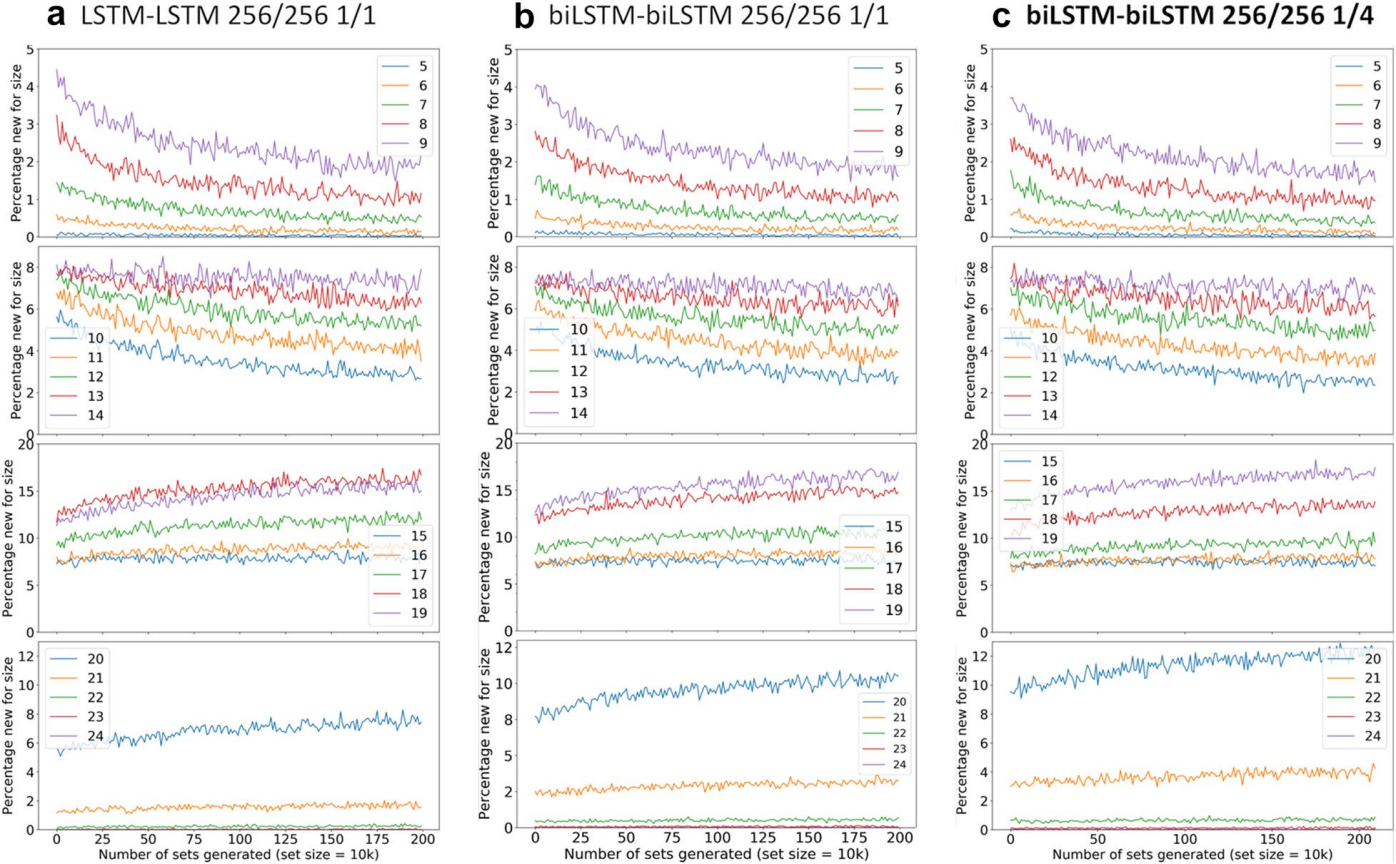
Novelty analysis by atom count. The winning architecture is highlighted in bold (See “Methods”)

For architectures A (LSTM–LSTM 256/256), B (biLSTM–biLSTM 256/256) and C (biLSTM–biLSTM 256/256 with 4 concatenated encoding layers), 200 sets of 10 k molecules were generated to create a total of 2M SMILES strings for each model. Every set of 10k molecules was considered a time point *t* in the analysis. For every set, all molecules were compared against all previously generated molecules and duplicates with the same InChIKey were excluded. The percentage of new molecules was subsequently expressed as number of new molecules divided by the number of valid molecules (Fig. 4a). All unique molecules were summed over time (Fig. 4b). An overall percentage of efficiency was expressed as number of valid unique molecules divided by the number of generated SMILES strings (Fig. 4b). The novelty was also analyzed by number of heavy atoms (Fig. 5).

### Size analysis of the training set

The impact of the size of the training set on the GENs was evaluated using random fragments subsets from PubChem [21], Zinc15 [39] and ChEMBL24 [40]. We have evaluated the sizes 225k, 45k and 9k with a random SMILES augmentation of five SMILES per molecule. The augmented smiles were deduplicated and the number of real augmentations may vary for each dataset. The datasets for ChEMBL and Zinc were subjected to the data preparation as described earlier for PubChem. The datasets were evaluated using architecture C with a biLSTM embedding layer of size 128 and 4 concatenated parallel biLSTM encoding layers of size 64. The datasets for ChEMBL and Zinc are available with the download of the source code [31].

## Results

We evaluated the performance of LSTM and GRU layers on architectures A and B (Table 1). For both architectures, the use of LSTM units led to higher percentages of valid SMILES strings and generated a very high percentage of valid SMILES (97%). All other computed metrics, i.e. percentage uniqueness and percentage training compounds, showed only minor fluctuations between the tested architectures. The use of GRU and biGRU layers consistently showed weaker results than architectures with LSTM or biLSTM layers. These architectures were discontinued in this study.

**Table 1:** Comparison architecture A and B and comparing LSTM to GRU

Architecture	Layer size	Best model epoch#	Validity%	Uniqueness%	Training%	Length match %^a^	HAC match %^b^
A: LSTM–LSTM	256/256	12, 17, 20	96.7 ± 0.4	99.9 ± 0.1	15.0 ± 0.7	98.2 ± 0.9	94.0 ± 1.8
A: GRU–GRU	256/256	15, 15, 15	91.8 ± 0.7	99.9 ± 0.1	12.6 ± 0.8	98.3 ± 0.4	94.6 ± 1.3
B: biLSTM–biLSTM	256/256	6, 7, 10	97.1 ± 0.4	99.9 ± 0.1	13.1 ± 0.5	98.2 ± 0.6	93.9 ± 0.8
B: biGRU–biGRU	256/256	11, 11, 11	95.6 ± 0.6	99.9 ± 0.1	15.0 ± 0.5	98.3 ± 0.3	93.1 ± 1.4

^a^Length match for SMILES length distributions of the training set and generated set (See “Methods”)

^b^HAC match for the atom count distributions of the generated set and training set (See “Methods”)

We extended our analysis to all four architectures A–D, followed by an evaluation using the same quality metrics (Table 2). Several important results were observed. Firstly, the increase of the layer size in architecture A and B led to a lower and more stable number of epochs needed to complete training. The use of larger RNN layers did not significantly improve the generative performance of the model. Indeed, the property match between the distributions of the generated set and the training set remained stable at 98% and 94% for SMILES length and HAC, respectively. Secondly, parallelization of the encoding layers (architecture C) provided a very good coverage of chemical space by generating a large number of new molecules. In particular, merging by concatenation improved the performance of the generative model. The match between the property distributions for SMILES length and HAC improved to 98.5% and 97.4%, respectively. Merging by averaging or a learnable weighted average moderately improved the results of the HAC match by 0.8–1.5% compared to the architecture B. These results suggest that merging by concatenation is preferred.

**Table 2:** Comparison architectures A, B, C and D

Architecture	Merge mode	Layer count	Layer size	Best model epoch#	Validity%	Uniqueness%	Training%	Length match%^a^	HAC match%^b^
A: LSTM–LSTM	–	1/1	64/64	54, 72, 63	95.4 ± 0.4	99.9 ± 0.1	12.0 ± 0.9	98.2 ± 0.3	94.0 ± 0.9
B: biLSTM–biLSTM	–	1/1	64/64	20, 22, 28	96.5 ± 0.5	99.9 ± 0.1	12.5 ± 0.9	97.9 ± 0.5	94.9 ± 0.8
A: LSTM–LSTM	–	1/1	256/256	17, 17, 20	96.7 ± 0.4	99.9 ± 0.1	15.0 ± 0.7	98.2 ± 0.9	94.0 ± 1.8
B: biLSTM–biLSTM	–	1/1	256/256	6, 7, 10	97.1 ± 0.4	99.9 ± 0.1	13.1 ± 0.5	98.2 +/0.6	93.9 ± 0. 8
*C: biLSTM–biLSTM*	*Concatenated*	*1*/*4*	*64*/*64*	*10, 14*, *16*	*97.0 ± 0.3*	*99.9 *±* 0.0*	*11.9 *±* 0.6*	*98.5 *±* 0.3*	*97.4 *±* 0.5*
C: biLSTM–biLSTM	Average	1/4	64/64	11, 15, 15	97.2 ± 0.3	99.9 ± 0.1	12.5 ± 0.3	98.6 ± 0.2	96.1 ± 0.7
C: biLSTM–biLSTM	Learnable average	1/4	64/64	15, 17, 23	97.6 ± 0.2	99.9 ± 0.0	14.6 ± 0.2	97.4 ± 0.4	94.8 ± 1.2
D: biLSTM–biLSTM	Concatenated	4/4	64/64	11, 11, 9	96.9 ± 0.3	99.9 ± 0.0	14.4 ± 0.5	97.4 ± 0.2	95.6 ± 1.2
D: biLSTM–biLSTM	Average	4/4	64/64	15, 17, 14	96.7 ± 0.1	99.9 ± 0.0	11.9 ± 0.2	98.1 ± 0.5	95.3 ± 1.1
D: biLSTM–biLSTM	Learnable average	4/4	64/64	12, 25, 18	95.6 ± 0.1	99.9 ± 0.0	10.4 ± 0.5	98.0 ± 0.2	96.2 ± 0.6
Influence of bidirectionality									
LSTM–LSTM	Concatenated	1/4	64/64	20,17,31	96.8 ± 0.4	99.9 ± 0.1	13.4 ± 0.5	97.6 ± 0.8	94.8 ± 1.3
biLSTM-LSTM	Concatenated	1/4	64/64	9, 14, 9	97.1 ± 0.3	99.9 ± 0.1	13.2 ± 0.5	97.7 ± 0.9	95.5 ± 1.4

Best architecture is highlighted in italics

^a^Length match for SMILES length distributions of the training set and generated set (See “Methods”)

^b^HAC match for the atom count distributions of the generated set and training set (See “Methods”)

Thirdly, architecture D with parallel embedding-encoding layers (architecture D) displayed moderately inferior results in comparison with architecture C (HAC−1.9%). However, the results were still moderately better than for architecture B (HAC +1.5%), suggesting the use of multiple parallel encoding layers is beneficial to train a stable generator. These results also suggested that a single bidirectional embedding layer was sufficient to describe the SMILES strings in the training space.

We have also tested two alternative architectures to better understand the importance of the bidirectional nature of the embedding and encoding layers (Table 2, last two lines). Modification of the embedding layer from LSTM to biLSTM significantly reduces the number of epochs needed to obtain a stable generator. Indeed, when comparing the architectures LSTM–LSTM and biLSTM–biLSTM (Table 2, last two lines), the number of epochs was decreased by half, from an average of 23 to 12 epochs. Introduction of bidirectional encoding layers improved the ability of the model to better reproduce the training space (HAC +1.9%). In conclusion, architecture C, a model based on a single bidirectional embedding layer followed by multiple concatenated bidirectional encoding layers, provided the best performance (in italics in Table 2).

Lastly, we evaluated the influence of the number of parallel-concatenated layers in architecture C (see Table 3). Increasing the number of parallel encoding layers reduced the number of epochs required to converge the generator. Parallelization simultaneously improved the ability of the generator to reproduce the property distribution of the training space. However, after reaching a plateau (at 4–5 layers) the introduction of new parallel layers did not further improve the model. Eventually a performance drop can be observed.

**Table 3:** Optimal number of parallel encoding layers in architecture C

Architecture	Merge mode	# Layers	Layer sizes	Best model epoch#	Validity%	Uniqueness%	Training%	Length match%^a^	HAC Match%^b^
B: biLSTM–biLSTM	–	1/1	64/64	20, 22, 28	97.1 ± 0.4	99.9 ± 0.1	13.1 ± 0.5	98.2 ± 0. 6	93.9 ± 0. 8
C: biLSTM–biLSTM	Concatenated	1/2	64/64	19, 19, 19	97.8 ± 0.4	99.9 ± 0.1	12.5 ± 0.4	97.3 ± 0.4	96.1 ± 0.1
C: biLSTM–biLSTM	Concatenated	1/3	64/64	12, 12, 12	97.2 ± 0.2	99.9 ± 0.0	12.2 ± 0.4	98.6 ± 0.3	96.9 ± 0.8
C: biLSTM–biLSTM	Concatenated	1/4	64/64	10, 14, 16	97.0 ± 0.3	99.9 ± 0.0	11.9 ± 0.6	98.5 ± 0.3	97.4 ± 0.5
C: biLSTM–biLSTM	Concatenated	1/5	64/64	8	95.9 ± 0.3	99.9 ± 0.0	13.5 ± 1.0	97.6 ± 0.2	97.2 ± 0.3
C: biLSTM–biLSTM	Concatenated	1/6	64/64	8	95.9 ± 0.2	99.9 ± 0.1	10.1 ± 0.4	96.3 ± 0.3	93.9 ± 0.7
C: biLSTM–biLSTM	Concatenated	1/7	64/64	7	96.8 ± 0.4	99.9 ± 0.0	14.0 ± 1.0	97.6 ± 0.6	95.9 ± 0.5
C: biLSTM–biLSTM	Concatenated	1/8	64/64	6, 6, 6	96.2 ± 0.7	99.9 ± 0.0	13.6 ± 0.1	98.0 ± 0.7	94.8 ± 0.8
C: biLSTM–biLSTM	Concatenated	1/16	64/64	5, 5, 5	95.9 ± 0.3	99.9 ± 0.0	13.5 ± 1.0	96.6 ± 0.7	93.1 ± 0.7

^a^Length match for SMILES length distributions of the training set and generated set (See “Methods”)

^b^HAC match for the atom count distributions of the generated set and training set (See “Methods”)

Overall, the best results were achieved with architecture C using one biLSTM embedding layer and 4 parallel concatenated biLSTM encoding layers. Using this architecture, an augmentation study was performed. Augmentation was done offline prior to training the network. Randomized SMILES were generated using RDKit by setting option *doRandom *= *True*, which was recently introduced to improve regression and classification models for physico-chemical properties [22, 23]. As expected, the augmentation improved the percentage of generated valid SMILES while lowering the number of training epochs. The performed analysis indicated that a fourfold augmentation provided the optimal result (Table 4). Additional augmentations only moderately improved the capability of the model to better reproduce the property space of the training set.

**Table 4:** Augmentation effect on architecture C biLSTM–biLSTM with layer sizes 64/64 and 4 concatenated encoding layers

Smiles	Augm.	Best model epoch#	Validity%	Uniqueness%	Training%	Length match%^a^	HAC match%^b^
Canonical	1	9, 9, 7	96.6 ± 0.5	99.9 ± 0.1	16.2 ± 1.5	93.3 ± 0.3	92.0 ± 0.5
Random	1	10, 14, 16	97.0 ± 0.3	99.9 ± 0.0	11.9 ± 0.6	98.5 ± 0.3	97.4 ± 0.5
Random	2	5, 5, 5	97.3 ± 0.1	99.9 ± 0.0	13.9 ± 0.5	97.7 ± 0.4	94.5 ± 0.8
Random	3	4, 6, 4	97.9 ± 0.3	99.9 ± 0.0	13.6 ± 0.5	98.8 ± 0.1	96.5 ± 0.2
*Random*	*4*	*4*, *3*, *4*	*98.2 *±* 0.4*	*99.9 *±* 0.0*	*11.6 ± 0.5*	*98.8 *±* 0.3*	*97.1 *±* 0.2*
Random	5	4, 4, 4	98.3 ± 0.3	99.9 ± 0.0	11.2 ± 0.5	97.3 ± 0.7	96.6 ± 0.3
*Random*	*10*	*4*, *4*, *4*	*98.3* ± *0.3*	*99.9 *± *0.0*	*14.2* ± *0.5*	*98.4 *±* 0.4*	*98.2 ± 0.5*

^a^Length match for SMILES length distributions of the training set and generated set (See “Methods”)

^b^HAC match for the atom count distributions of the generated set and training set (See “Methods”)

After selection of the best architecture (C/BiLSTM–BiLSTM/256-256/4 concatenated), we generated 2M SMILES strings. We computed 12 molecular properties (See “Methods”) at the beginning and end of the generation process. The computed distributions were compared to the distributions of the training space (Fig. 3). The distributions for the generated sets show a strong match to the distributions of the training set. As expected, we observed a shift for all distributions correlated to molecular size. This observation suggests that the generator starts to saturate the chemical space of smaller molecules with increasing number of generated molecules. Indeed, the distributions of molecules with sizes 5 and 6 were close to 0 after generation of 250k and 1M SMILES, respectively. The reduced error observed for the Jensen-Shannon divergence on all distributions suggested that the property distributions of the created SMILES were stable after 2M generated SMILES (Table 5).

**Table 5:** Percentage match measured as continuous Tanimoto (Tan; Eq. 1) or Jensen-Shannon Divergence (JSD; Eq. 2) between the distributions of the training space and generated compounds at early (10k) and late stage (2M) generation

	Tan 10k	Tan 2M	JSD 10k	JSD 2M
Size:				
SMILES length	94.1 ± 0.4	84.6 ± 0.1	0.170 ± 0.004	0.252 ± 0.000
Heavy atom count (HAC)	98.8 ± 0.2	94.1 ± 0.1	0.058 ± 0.004	0.142 ± 0.000
Molecular Weight (MW)	97.4 ± 0.2	92.7 ± 0.1	0.124 ± 0.002	0.187 ± 0.000
Polarity:				
logP	99.6 ± 0.0	99.1 ± 0.0	0.042 ± 0.002	0.055 ± 0.001
TPSA	99.6 ± 0.1	95.7 ± 0.1	0.044 ± 0.001	0.097 ± 0.000
Topology:				
Rotatable bond count	99.5 ± 0.1	96.5 ± 0.0	0.042 ± 0.002	0.099 ± 0.001
Fraction cyclic	99.2 ± 0.2	95.6 ± 0.1	0.051 ± 0.002	0.106 ± 0.000
Fraction conjugated	99.6 ± 0.1	99.7 ± 0.1	0.047 ± 0.003	0.084 ± 0.000
Fraction aromatic	99.7 ± 0.1	99.5 ± 0.1	0.060 ± 0.002	0.109 ± 0.001
Composition:				
Fraction carbon	98.6 ± 0.2	97.0 ± 0.0	0.061 ± 0.003	0.106 ± 0.000
Fraction nitrogen	99.6 ± 0.2	96.1 ± 0.1	0.097 ± 0.004	0.132 ± 0.000
Fraction oxygen	99.4 ± 0.1	99.4 ± 0.1	0.050 ± 0.003	0.058 ± 0.001

We have also analyzed the ability of the models to generate new molecules. For all datasets, the number of new molecules decreases slowly over time. The novelty rates after generating 2 M compounds are 66.3, 73.3% and 75.4% for architectures A (LSTM–LSTM 256/256), B (biLSTM–biLSTM 256/256) and C (biLSTM–biLSTM 256/256 1/4), respectively. We observed further that the novelty rate for architecture A was systematically lower than the novelty rate for architectures B and C. These results suggest that the use of bidirectional LSTM units is beneficial to maintain a high degree of generativity for the trained model. We also evaluated the total number of generated molecules over time (Fig. 4b). The generation efficiency of the model was as percentage of valid unique molecules. After 2M, architecture A with two consecutive LSTM–LSTM produced 1470,543 unique molecules with an efficiency of 73.5%. Architectures B and C with bidirectional embedding and encoding layers have generated 1566,535 (78.5% efficiency) and 1602,018 (80.1% efficiency) unique molecules, respectively. The use of bidirectional layers was thus highly beneficial to improve the efficiency of the generation process.

As expected, the novelty decreased over time as a result of saturation of the chemical space and gradually moves from smaller to larger molecules (Fig. 5). Additionally, the results for the novelty rate showed a subtle shift when moving from architecture A to architectures B and C. These results are in line with earlier observations that bidirectional RNN layers improve the performance of the deep generative models.

**Fig. 6:**
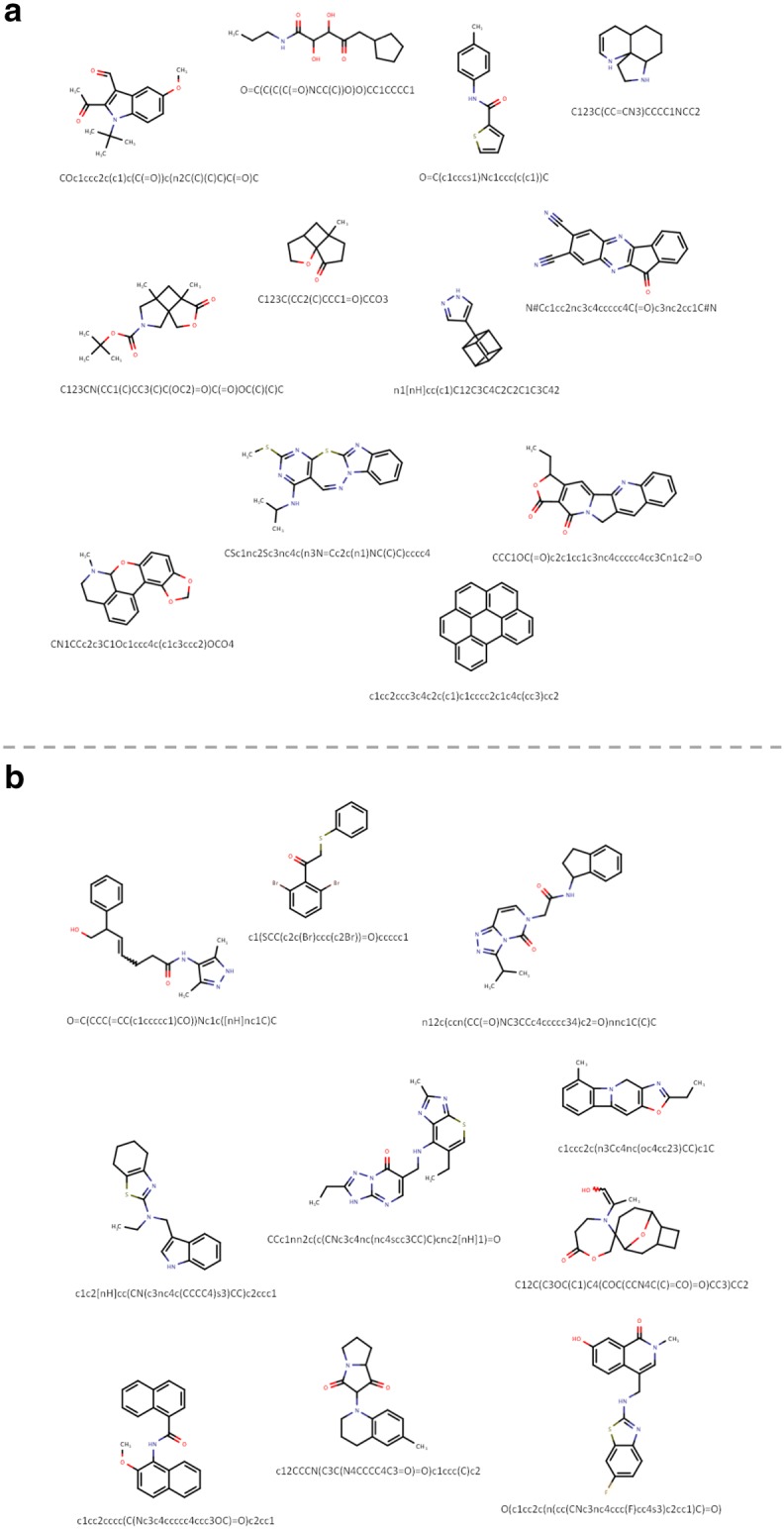
Generated SMILES strings for Selected examples from ChEMBL24 and Zinc evaluated in Table 6. **a** 12 molecules generated by the model after training with 225k randomly selected ChEMBL24 molecules. **b** 10 selected examples of SMILES generated after training the model with 225k randomly selected molecules from Zinc15

Lastly, we measured the impact of the training set size on the performance of the GENs. This evaluation was done to investigate whether a well-defined architecture could autonomously learn the alphabet and grammar of the SMILES strings without the need for didactic feedback from either a discriminator or through reinforcement learning. In this comparison we tested models developed with PubChem, ChEMBL 24 and Zinc15 datasets using 9k, 45k and 225k unique molecules (Table 6). The results for all datasets showed clear improvements for the number of valid molecules and moderate improvements for the percentage match of heavy atom count with increasing training set size. Indeed, the percentage of valid molecules for PubChem increased from 81.3 to 98.3%, from 74.2 to 94.6% for ChEMBL and from 77.2 to 95.2% for Zinc. The small differences between the datasets was expected and can be explained by the fact that we used a focused library for the PubChem dataset while the training sets of ChEMBL and Zinc were selected by chance. Measures for other properties are relatively stable with increasing size. These results confirm the hypothesis that a large training set is beneficial to learn the SMILES alphabet and grammar of the training data. Large datasets also significantly shortened the number of epochs needed to train a GEN. Examples of generated molecules for the models trained with ChEMBL 24 and Zinc15 are displayed in Fig. 6a, b, respectively. The small set of selected examples clearly demonstrates that the autonomously learning generator can easily handle complex SMILES and generates SMILES with a vivid curiosity and open-mindedness. Consequently, the generators are well equipped to explore new areas of chemical space.

**Table 6:** Impact of the training set size on GENs performance

Dataset and evaluated size	Augmented size with real factor^a^	Best model epoch #	Validity%	Uniqueness%	Training%	Length match%^b^	HAC match%^c^
PubChem225k							
9k	54,624 (4.8)	10, 10, 10	81.3 ± 0.9	100.0 ± 0.0	0.3 ± 0.1	97.7 ± 0.0	90.5 ± 0.0
45k	218,124 (4.8)	5, 5, 5	95.6 ± 0.7	99.9 ± 0.1	2.6 ± 0.5	99.0 ± 0.0	94.7 ± 0.0
225k	1088,864 (4.8)	4, 4, 4	98.3 ± 0.3	99.9 ± 0.0	11.2 ± 0.5	97.3 ± 0.7	96.6 ± 0.3
Chembl24							
9k	35,928 (4.0)	44, 43, 45	74.2 ± 1.9	99.0 ± 0.2	0.2 ± 0.2	81.9 ± 5.4	95.9 ± 1.0
45k	179,888 (4.0)	5, 6, 5	91.9 ± 1.9	100.0 ± 0.0	0.2 ± 0.1	90.6 ± 2.8	97.6 ± 1.4
225k	896,214 (4.0)	9, 6, 6	94.6 ± 0.1	100.0 ± 0.0	1.4 ± 0.3	88.4 ± 1.6	98.1 ± 0.6
Zinc15							
9k	32,546 (3.6)	24, 21, 21	77.2 ± 1.0	100.0 ± 0.0	0.0 ± 0.0	82.2 ± 3.3	91.2 ± 1.1
45k	163,929 (3.6)	10, 7, 11	90.4 ± 1.1	100.0 ± 0.0	0.1 ± 0.1	87.6 ± 1.2	92.6 ± 1.1
225k	820,747 (3.6)	4, 6, 6	95.2 ± 0.3	100.0 ± 0.0	0.3 ± 0.1	90.4 ± 1.2	93.5 ± 1.2

^a^Size of the augmented dataset after 5 random attempts per SMILES and de-duplication to unique SMILES. Real augmentation factor varies depending on dataset

^b^Length match for SMILES length distributions of the training set and generated set (See “Methods”)

^c^HAC match for the atom count distributions of the generated set and training set (See “Methods”)

## Discussion

Our goal was to obtain powerful SMILES based molecular generator, which can generate a high degree of valid novel molecules within the property space of the training set. To obtain such a generator, we modified an earlier LSTM-based architecture [6]. Key modification is the introduction of an EarlyStopping mechanism with an online generator used to perform a statistical quality control at every epoch. This new feature allowed us to better monitor the learning process of the generator and to apply an overfit control [20]. Using this method, we can now select the earliest stable model capable of generating a very high percentage of valid molecules. Indeed, within a few training epochs, we reached very high percentages of valid SMILES produced by the generator (95.9–98.3%). Our analysis showed that the decision to stop training early based on the percentage of valid molecules, did not affect the capability of the model to generate SMILES with a high degree of novelty. The use of this EarlyStopping-mechanism may also be beneficial for use with RNN-based predictive models to partially freeze layers from further optimization.

Selection of LSTM in architecture with parallel encoding layers, allowed us to reduce the number of hyperparameters in the network, while maintaining a stable generator with excellent generation results. We also explored the merging of the parallel layers using concatenation, averaging or by learnable average. The results were clearly in favor of concatenation. A comparison between the results for architectures C and D shows that a single biLSTM embedding layer is sufficient to describe the training set.

Using the best architecture, we tested the effect of SMILES augmentation to further improve the ability of the model to generate a higher percentage of valid molecules and/or better reconstruct the property space. Our results demonstrated that augmentation increased the percentage of valid molecules from 97.0 to 98.3%. The models developed using high augmentation provided nearly perfect reconstruction of the property space (97.1 to 98.2%). Increasing augmentation affected, however, the novelty rate in the generated molecules.

The herein introduced GENs generated SMILES strings with equal or better quality when compared to the recently published RNN-based SMILES generator [7, 14, 25, 41]. Contrary to the earlier work, GENs reached these results using a significantly smaller training sets and a small number of training epochs. Using an examination mechanism as EarlyStopping method [20] thus proves highly advantageous to control the training of deep generative models.

In a typical GAN architecture two networks compete, i.e. a Generator and a Discriminant. To our knowledge the closest method to our GEN is SeqGAN [42]. SeqGAN is modeling the generator as a stochastic policy. The reward signal coming from the discriminator is judged on a complete sequence, and gradient is passed back to the intermediate state-action steps using Monte Carlo search. Recent introduction of the Wasserstein distance in GAN (WGAN) improves the generator [43, 44] by using a smooth metric for measuring the distance between two probability distributions.

In GENs, a discriminator is absent and is replaced by an independent examiner. The examiner applies a statistical assessment on the quality of the generator output after every epoch. GANs typically need full datasets to perform a sound evaluation. The proposed examination mechanism is based on a single generative model for SMILES string generation. Its generator mechanism is autonomously learning the training set and it is not influenced by the feedback from the examiner. This also differentiates GENs from GANs or models with reinforcement learning (RL), which both require a feedback mechanism. Nevertheless, as demonstrated in this work, GENs achieve spectacular results on the reconstruction of the chemical space of the training set with a vivid curiosity and open-mindedness. The latter is expected to be the result of the GEN methodology allowing the generator to acquire the knowledge by self-directed learning while being independently examined and stopped as soon as it has acquired a sound level of knowledge [20].

GENs are open to accommodate any neural network and early-stopping mechanism and can be used for other modeling questions. Additionally, training can be easily continued in GENs and are thus open to transfer learning (TL) [45].

If we treat SMILES as text, one can notice that SMILES contain two major graph conversion challenges, ring and branch representation in 1D. They can be considered as analogs of grammar and conjugation in natural languages. However, SMILES only contain a small amount of unique characters, i.e. chemical “words”. Based on the excellent results we observed using GENs, we believe that this limiting number of words, can be deciphered very quickly by neural networks when selecting an appropriate architecture. Moreover, over-training deep generative models will even lead to the loss of the novelty of generated structures. The latter problem is also a classical issue known to GANs. In our opinion, a generator should learn the domain space but at the same time it must also have sufficient freedom to apply the extracted rules and maintain diversity of generated answers. The latter has been proposed by introducing the examination mechanism in GENs. The optimal examination mechanism in GEN needs to be defined and fine-tuned on a case-by-case basis. It is important to highlight, that the independent quality mechanism introduced in this work does not influence the generator. In summary, the introduced GENs are a welcome addition to recent developments in artificial intelligence (AI). GEN can learn by itself and its ability to generalize the knowledge is checked by a quality test. This is very similar to IBM’s Watson [46] passing the physician exams, proving that AI can acquire the same level of knowledge as any other Homo Sapiens student of a college (http://ibm.com/watson).

## Conclusion

The main goal of a generator is to produce a set of SMILES with a high degree of novelty while staying focused on the property space of the training set. By small adjustments to an existing architecture we obtained remarkable results for both these goals. Our GENs autonomously learn the alphabet and grammar of SMILES strings to generate valid molecular SMILES within the property space defined by the training set. The examination mechanism allows us to stop training after a few epochs. The winning architecture used an ensemble of smaller networks, capable of achieving similar results as a large network [47]. The analysis of different architectures showed that the use of a bidirectional embedding layer followed by multiple parallel encoding layers is essential for stable generation results. SMILES augmentation increased the volume of the training set and accuracy of produced models without the need for a larger set of diverse molecules. In this study we analyzed performance of global generation models by focusing on their validity, novelty and coverage of the generators. The proposed approach can be also used via transfer learning to generate compounds for specific scaffolds (see e.g. [14–17]).

The introduced early-stopping mechanism of the generators allows maintaining a high degree of novelty thanks to online statistical quality control, measuring the percentage of valid SMILES. The EarlyStopping mechanism is easily adaptable and open to accommodate other quality metrics such as distribution overlap, multi-objective targets or other models. GENs can thus be easily adapted to address other tasks. After EarlyStopping, training of the GENs can be continued to tackle new challenges [45]. The code including example notebooks is distributed freely [31] under a Clause-3 BSD License (https://opensource.org/licenses/BSD-3-Clause).

## Data Availability

The written code and public datasets used for this research is available on https://github.com/RuudFirsa/Smiles-GEN.
